# Sorting through the impact of familiarity when processing vocal identity: Results from a voice sorting task

**DOI:** 10.1177/1747021819888064

**Published:** 2019-11-22

**Authors:** Sarah V Stevenage, Ashley E Symons, Abi Fletcher, Chantelle Coen

**Affiliations:** School of Psychology, University of Southampton, Southampton, UK

**Keywords:** Familiarity, vocal identity processing, voice sorting task

## Abstract

The present article reports on one experiment designed to examine the importance of familiarity when processing vocal identity. A voice sorting task was used with participants who were either personally familiar or unfamiliar with three speakers. The results suggested that familiarity supported both an ability to tell different instances of the same voice together, and to tell similar instances of different voices apart. In addition, the results suggested differences between the three speakers in terms of the extent to which they were confusable, underlining the importance of vocal characteristics and stimulus selection within behavioural tasks. The results are discussed with reference to existing debates regarding the nature of stored representations as familiarity develops, and the difficulty when processing voices over faces more generally.

In recent years, there has been a growing interest in the field of voice processing. Voices represent an important and informative social cue so much so that they have been described by Belin, Fecteau, and Bédard (2004) as “auditory faces.” They reveal information about what someone is saying (speech), how someone is feeling (affect), and of course who someone is (identity). Furthermore, like faces, the starting assumption has been that voices are such an important cue to identity that their processing should be strong. However, evidence comparing the processing of faces and voices has challenged this assumption: When trying to recognise an individual, voice processing is easily affected by emotional change (Read & Craik, 1995), clip length (Cook & Wilding, 1997; Yarmey & Matthys, 1992), interference (Stevenage et al., 2013), and delay (Papcun, Kreiman, & Davis, 1989; Saslove & Yarmey, 1980; Yarmey & Matthys, 1992). Moreover, relative to faces, voices more often elicit a familiar-only state in which someone is recognised as familiar but cannot be placed (Hanley, Smith, & Hadfield, 1998).

The processing of voices has arguably been shown to be even weaker when voices are *unfamiliar* than when familiar (see Stevenage, 2018, for a review). At a general level, unfamiliar voices elicit slower sex judgements (Burton & Bonner, 2004), difficulty during speech shadowing or comprehension (Johnsrude et al., 2013; Kreitewolf, Gaudrain, & von Kriegstein, 2014; Levi, Winters, & Pisoni, 2011; Nygaard, Sommers, & Pisoni, 1994; Souza, Gehani, Wright, & McCloy, 2013), and weaker event-related potential (ERP) waveforms during expression judgements (Pinheiro et al., 2016). When processing vocal identity, unfamiliar voices also fail to demonstrate the usual facilitation effects when presented in synchrony or near-synchrony with their corresponding face (Gonzalez et al., 2011; Schweinberger, Kawahara, Simpson, Skuk, & Zäske, 2014), and they show no benefit through repetition priming following prior presentation of the corresponding face (Ellis, Jones, & Mosdell, 1997; Schweinberger, Herholz, & Stief, 1997; Schweinberger, Robertson, & Kaufmann, 2007; Stevenage, Hugill, & Lewis, 2012) or voice (Schweinberger, 2001) (see Bülthoff & Newell, 2017). Finally, performance in a familiar voice recognition task shows no association with performance in an unfamiliar voice discrimination task (see Cook & Wilding, 1997; van Lancker & Kreiman, 1987, Supplementary Materials). While stimuli and task demands differ in these tasks, and suggest caution in overinterpreting these data, the lack of association also suggests that familiar and unfamiliar voice processing may differ because they rely on quite different mechanisms.

These differences have fuelled a number of studies which have highlighted a fundamental distinction in the processing of familiar and unfamiliar voices. This distinction echoes a similar discussion in the domain of faces, in which participants’ capacity for familiar and unfamiliar face recognition has also been determined to be independent of one another (Megreya & Burton, 2006). Of more relevance, a distinction between familiar and unfamiliar voice processing is supported by evidence of neural separation: Familiar voice recognition depends on activation of anterior parts of the superior temporal sulcus (STS) and superior temporal gyrus (STG), whereas unfamiliar voice recognition depends on activation of more posterior parts of these regions (Belin & Zatorre, 2003; Bethmann, Scheich, & Brechmann, 2012; von Kriegstein, Eger, Kleinschmidt, & Giraud, 2003; von Kriegstein & Giraud, 2004; Warren, Scott, Price, & Griffiths, 2006).

All differences highlighted so far may be explained by the fact that familiar voices have a preexisting mental representation. Without this, a voice may only be processed on the basis of a piecemeal analysis of rather superficial vocal characteristics (Kreiman & Sidtis, 2011) leading to an impoverished manner of processing (Lavan, Burton, Scott, & McGettigan, 2019). In contrast, the existence of a preexisting mental representation serves as a point of comparison when processing a familiar voice, and may enable a listener to solve two particular problems—to map together different instances of the same person, and to map apart similar instances of two different people (see Young & Burton, 2017, for an overview of this discussion in the face domain). This pattern of performance implies that the mental representation for a familiar voice may capture information not only about the differences between two speakers but also about the natural variation within a single speaker. It would also predict that familiarity with a speaker may serve as a protective factor when recognising voice clips that vary naturally rather than being too constrained or controlled.

In this regard, a number of studies now exist which contribute to this issue. First, Lavan, Scott, and McGettigan (2016) presented two studies in which speaker discrimination was tested across clips depicting vowel sounds, volitional laughter, and spontaneous laughter. The results suggested that a change in vocalisation (speech to laughter) and a change from volitional to more spontaneous vocalisations, both led to difficulty during a speaker discrimination task. In other words, it was hard to generalise identity cues across the different types of speaker clips, suggesting that vocal variability represented a challenge. Importantly, although personally familiar listeners performed better than unfamiliar listeners (Experiment 2), both listener groups were equally affected by vocal variety suggesting that familiarity did not confer an advantage as might have been predicted.

In contrast, the results of a sorting task suggested a different picture. Borrowing from the face domain, a sorting task involves the presentation of instances of two or more identities, with these instances allowed to vary naturally through the use of what Jenkins, White, van Montfort, and Burton (2011) called “ambient stimuli.” The participant’s task is to sort the ambient stimuli into clusters to reflect the number of identities they perceive. Correctly grouping the instances for one identity into a single cluster represents the ability to successfully map different instances of the same person together. Conversely, mixing identities within a single cluster represents a failure when mapping similar instances of two different people apart. In this way, the sorting task presents a simple yet powerful methodology which successfully separates out these two aspects of recognition. Moreover, it is a task which can be undertaken regardless of one’s familiarity with the stimuli, allowing task demands to be held constant.

When the sorting task was used with faces (Jenkins et al., 2011), the results suggested a difference in the processing of familiar and unfamiliar perceivers. Both groups were able to tell faces apart (producing few mixed-identity clusters). However, familiarity significantly enabled participants to tell faces together such that the familiar perceivers accurately sorted the instances into fewer identity clusters than the unfamiliar perceivers. These results have since been replicated (Andrews, Jenkins, Cursiter, & Burton, 2015; Redfern & Benton, 2017; Zhou & Mondloch, 2016) suggesting a robust benefit of familiarity when coping with natural variation to tell different instances of the same person together.

When the same task was applied in the voice domain, the results were remarkably consistent to those obtained with faces. Lavan et al. (2018) used three pairs of female voices from the TV series “Orange is the New Black.” Familiarity was manipulated by recruiting participants who had either watched the TV series or who had not. As with faces, the task was to sort speaker instances into clusters to reflect the number of identities that the participant perceived, and performance was evaluated in terms of the ability to tell speakers together and to tell speakers apart. Overall, the results replicated the pattern obtained with faces. Familiarity better enabled listeners to tell voices together without affecting their ability to tell voices apart. Moreover, the results were replicated using a different set of stimuli from the TV series Breaking Bad (Lavan, Burston, et al., 2019), both when stimuli portrayed low expressiveness (i.e., neutral speech clips) and when they portrayed high expressiveness (i.e., shouting or strained speech clips). As such, the sorting task has elicited a robust and consistent pattern of performance across (faces and) voices suggesting that familiarity helps listeners to tell instances from the same identity together.

Two aspects of the current findings warrant some consideration. First, putting the results of Lavan et al. (2016) alongside those of the voice sorting task highlights a contradiction. The results of the voice discrimination task suggested that familiar listeners performed better overall but were as affected as unfamiliar listeners by vocal variability. In contrast, the results of the sorting task suggested that familiar listeners were less affected than unfamiliar listeners by vocal variability and thus were better able to tell instances of the same voice together. This contradiction may have arisen through the use of stimuli which differed both in their nature, and in their basis for familiarity. Specifically, the voice discrimination task used stimuli that consisted of vowels or laughter clips, and that were personally familiar, whereas the sorting task used stimuli that were speech-based and were publicly familiar (famous celebrities). The change from vowels and laughter clips to speech-based clips arguably enabled the use of stimuli that were richer in both duration and vocal variety. Consequently, familiarly may have conferred more of an advantage when using speech clips because there was more vocal variety for listeners to cope with. Similarly, the change from personally familiar to celebrity voices may have reflected a change in the fundamental nature of the stored mental representation that a listener could draw on. Personally familiar stimuli may be represented by a richer representation capturing the vocal variety that a listener experiences through personal contact. On the contrary, celebrity stimuli may be represented by a weaker representation given either less exposure to celebrity voices than faces (see Barsics & Brédart, 2011; Brédart, Barsics, & Hanley, 2009), or less exposure to the full range of listening conditions, or intraspeaker variations that characterise human speech. In the face domain, this has led researchers to suggest that we may process celebrity stimuli in quite a different way to personally familiar stimuli (Carbon, 2008; Wiese et al., 2018) and there is no empirical reason to suggest that this may not also be the case in the voice domain. With this in mind, the natural next step is to use personally familiar speech-based stimuli within the sorting task to see whether the previous pattern of results is replicated. This is the primary purpose of the current study.

The second aspect that warrants consideration rests on the existence of subtle item effects within Lavan, Burston, and Garrido’s (2018) sorting task. Specifically, one of the three pairs of voices tested (Set 1) could be grouped together with equal ease across familiar and unfamiliar listeners, as judged by the number of clusters created. Yet, this pair revealed more “telling apart” errors for unfamiliar than familiar listeners, as shown through a greater number of mistakes when grouping voices together. The authors tentatively suggested a role for either speaker distinctiveness or speaker variability in accounting for these item effects, but an analysis of vocal characteristics including valence, arousal, pitch, and apparent vocal tract length did not help in understanding the observed item effects. Given Burton’s (2013) observation that the very pattern of variability within an identity may be a cue to identity in itself, a second aim of the present study was to provide a more detailed examination of potential item effects.

The present study used a voice sorting task with listeners who were either personally familiar or unfamiliar with a set of voices. Rather than using two voices as in previous studies, the use of three voices here provided a more ecologically valid sorting task by increasing the variability of the voice clips within the sorting set. The impact of familiarity was examined both when telling voices together and when telling voices apart. On the basis of previous evidence, it was predicted that familiarity would help listeners to tell clips of the same identity together. However, it was predicted that there would be no significant benefit when telling identities apart given a low likely incidence of confusion errors. In addition, an analysis of vocal characteristics was presented across the three speakers, with the prediction that performance on the sorting task may be linked to the degree to which each speaker in the set vocally stood out from the others.

## Method

### Design

A voice sorting task was used in which participants were asked to sort a set of voice clips into identity clusters. Critically, and unbeknownst to them, participants were either personally familiar or unfamiliar with the speakers providing the clips. Dependent variables included the number of identity clusters following sorting, the number of “intrusion errors” within each cluster, and the self-rated confidence in the solution. In addition, measures of within-speaker clustering, and cross-speaker mistakes were examined, together with a misidentification index for each voice clip.

### Participants

A total of 45 participants (26 females, 19 males) took part in the present study, either on a volunteer basis or in return for course credit. Of these, 22 participants (13 females) were “familiar” with the speakers, in that they had either been taught by all speakers, or were colleagues within the same research group. Teaching contact took the form of twice-weekly lectures across a minimum of 5 weeks within 1 to 3 months of participation, supplemented by tutorials, informal conversational exchanges, and access to audio recordings provided by the speakers as support for teaching. As such, the familiar group of participants were deemed to be familiar with the speakers in terms of recency, breadth, and depth of interaction. In contrast, the remaining 23 participants (13 females) were “unfamiliar” with the speakers in that they did not know, and had not been taught by, any of the speakers.

Participants varied in age from 18 to 29 years (*M* = 22.02, *SD* = 4.36) minimising the risk of age-related hearing loss. In addition, all were native English speakers, or had lived in the United Kingdom for at least 7 years, removing the potential for speech comprehension difficulties.

### Materials

The stimuli for the present study comprised 52 speech clips as read by three female Caucasian members of the Psychology Teaching Staff. The speakers were aged 36, 44, and 49 years at the time of recording and all were nonsmokers. All spoke English as a first language and had a British accent which varied slightly in regional vowel sounds.

The speech clips consisted of excerpts from Mr Tickle© (Hargreaves, 1971) which were taken from a complete reading of the Mr Tickle extract as used in the British Library “Your Voices” project on regional and national accents (http://www.bl.uk/learning/langlit/sounds/your-voices/your-accent/). All three speakers provided a complete recording of the extract, providing natural variation in intonation across the extract “as if reading to a small child.” Speech was captured on an Olympus VN-541PC Digital Voice Recorder, with 4 GB flash memory, set to record in “memo” mode with a low-cut filter providing noise cancellation. Variation in ambient noise was minimised through all recordings being obtained in the same quiet recording room.

From these complete extracts, Audacity 2.1.0 was used to obtain 52 speech clips for each speaker reflecting self-contained sentences or phrases. These ranged in length from 0.9 to 6.34 s. From these three sets of 52 speech clips, 19 clips were selected from Speaker A, 17 clips were selected from Speaker B, and 16 clips were selected from Speaker C such that each clip was spoken by only one speaker, and all clips together comprised the entire Mr Tickle extract. The use of an unequal number of clips from each speaker was purposeful, and sought to work against any task demands in which participants may assume a need to form identity clusters of equal sizes. The remaining speech clips were discarded.

In addition to these Mr Tickle speech clips, the voices of six unfamiliar male speakers provided 16 speech samples for use in a practice trial. These were all drawn from the SuperIdentity stimulus database, and each sample consisted of the speaker uttering one of several scripted phrases. The 16 samples comprised four phrases from Speaker 1, four phrases from Speaker 2, three phrases from Speaker 3, two phrases from Speaker 4, two phrases from Speaker 5, and one phrase from Speaker 6. Again, the use of an unequal number of clips from each speaker here sought to minimise any task demands to create identity clusters of equal sizes.

Finally, a pre-experimental questionnaire was prepared which took the form of a paper-based familiarity rating task. The names and faces of 18 University staff members were depicted alongside a familiarity rating scale ranging from 1 (*not at all familiar*) to 7 (*highly familiar*). The staff members included men and women drawn from psychology and nonpsychology staff. Critically, the three psychology lecturers who provided the speech clips were included in this questionnaire which thus served both as a familiarity rating task, and as a priming mechanism to reduce the risk of a “tip of the tongue” state.

Experimental stimuli were presented, and data were recorded using PowerPoint running in edit mode rather than slideshow mode so that the participants could interact with the stimuli on each slide. Written instructions were embedded within the PowerPoint slides and were displayed on the 13ʺ colour monitor attached to a MacBook Air laptop running OS X El Capitan (Version 10.11.6). Sound was presented via the computer speakers with the volume preset at a comfortable but adjustable level.

### Procedure

Following the provision of informed consent, participants were tested individually within a quiet research cubicle. Before the voice sorting task began, participants were first asked to complete the familiarity rating questionnaire. This was presented as an unrelated task. In actual fact, the rating questionnaire allowed the experimenters to prime the participants to the identity of the three speakers and to obtain a familiarity rating for each speaker. On the basis of these ratings, the assignment of participants to the “familiar” and “unfamiliar” groups was verified.

Following the familiarity rating task, participants were introduced to the experimental task. This was described as a voice sorting task, with the method mimicking the free-sort face task used by Jenkins et al. (2011) and Andrews et al. (2015) and used more recently with voices by Lavan, Burston, and Garrido (2018) and Lavan, Burston, et al. (2019) (see also Lavan, Merriman, et al., 2019). Participants were instructed that they would be presented with a set of voice clips, which appeared as loudspeaker icons on a PowerPoint slide. Clicking on each loudspeaker icon enabled each recording to be played. Their task was to listen to each voice clip, and then drag them to form an unspecified number of identity clusters such that all the clips within one cluster would represent one speaker, and all the clips within another cluster would represent another speaker. As such, the participants were instructed that the number of clusters left on the slide at the end of the process would reflect the number of speakers that they felt were present across the set of clips. Participants were encouraged to listen to each clip as many times as they wished, and were shown how to adjust the playback volume. Finally, they were asked to indicate their confidence in their final solution by dragging a number from 1 (*not at all confident*) to 7 (*very confident indeed*) from the onscreen display into a marked “confidence” box.

After the opportunity to ask any clarifying questions, participants completed one practice trial with the 16 unfamiliar male speakers. This enabled participants to get used to the format of the task, and feedback was given on their performance by revealing the true number of practice trial identities. The practice trial also enabled participants to appreciate that there could be an unequal number of instances of each speaker, and participants were able to reflect privately on their strategy and their accuracy prior to the main trial.

**Figure 1.**
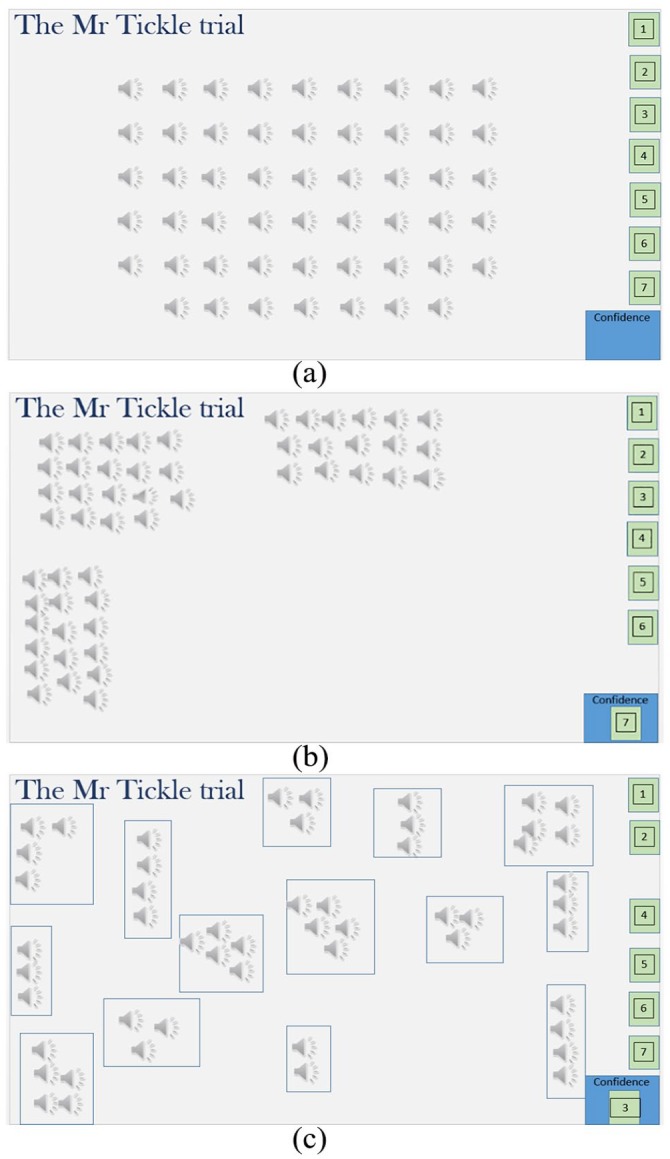
The starting point of the Mr Tickle trial (a) together with the 3-identity solution of a familiar listener (b), and the 14-identity solution of an unfamiliar listener (c).

Following the practice trial, participants completed the main Mr Tickle trial which involved sorting the 52 clips that made up the Mr Tickle excerpt. These were initially arranged in a fixed-random order rather than in a sequential story order to minimise any perception that one clip may flow into the next either semantically, or in terms of speaker identity. Participants dragged the Mr Tickle clips to form identity clusters, and indicated their confidence in their solution as before (see Figure 1).

Finally, participants were asked whether they spontaneously recognised, and could name, any of the speakers in the Mr Tickle trial. If they could not spontaneously name the speakers, one final clip of each speaker was available, in which they all uttered the same scripted phrase. Participants were asked to type a name, or other identifying information, into a box beneath each loudspeaker icon to indicate the identity of the speakers. Together, the spontaneous naming task and the cued naming task served as a final test of familiarity with the speakers’ voices. The entire procedure lasted approximately 45 min, after which participants were thanked and debriefed.

## Results

Prior to analysis, participant familiarity with the three speakers was examined through both the pre-experimental rating task, and the post-experimental spontaneous or cued naming tasks. In terms of familiarity ratings, two participants indicated familiarity with only one of the three speakers. At the post-experimental stage, only that one speaker elicited either a name or unique identifying information either spontaneously or when cued with the additional clip as a prompt. These two listeners were quite different from the remaining participants in the familiar group who gave ratings of 4 or more for all speakers, and who gave a positive identification by name or by unique identifying information at the post-experimental stage. The two participants who failed to reach these strict criteria were dropped from all subsequent analyses, leaving 20 participants in the personally familiar group, and 23 participants in the unfamiliar group.

Given that the familiarity ratings were not normally distributed for both listener groups according to Shapiro–Wilk tests (both *p*s < .021), a Mann–Whitney *U* test was used to check the difference in speaker familiarity across the two groups. As anticipated, this confirmed that the familiar group (*M* = 5.95, *SD* = .85, Median = 6, Mode = 7) did indeed show a significantly higher rated familiarity with the speakers than the unfamiliar group (*M* = 1.54, *SD* = 1.23, Median = 1, Mode = 1; *U* *=* 5.59, *p* < .001).

With this established, performance on the voice sorting task was assessed by means of a number of dependent measures. First, the number of identity clusters and the number of “intrusion errors” were calculated as in Jenkins et al.’s (2011) face sorting task. These provided overall measures indicative of telling together and telling apart for the familiar and unfamiliar listeners alike. Second, the matrices of within-identity performance and the cross-identity performance were calculated as per Lavan et al.’s (2018; Lavan, Burston, et al., 2019) voice sorting tasks. These provided more nuanced measures of telling together and telling apart which could be separated by speaker identity. Third, a misidentification index was calculated as per Lavan, Burston, et al.’s (2019) voice sorting task. This provided a single score per voice clip which combined “telling together” and “telling apart” to represent the extent of confusability for each clip taken individually. Finally, self-rated confidence in the solution was examined, providing a metacognitive measure of performance alongside the behavioural measures above. Shapiro–Wilk tests for all measures within each listener group indicated that the data were not normally distributed (*p* < .05 for all measures). Consequently, nonparametric tests were used both when exploring familiarity effects and item effects.

### Number of identity clusters

**Figure 2.**
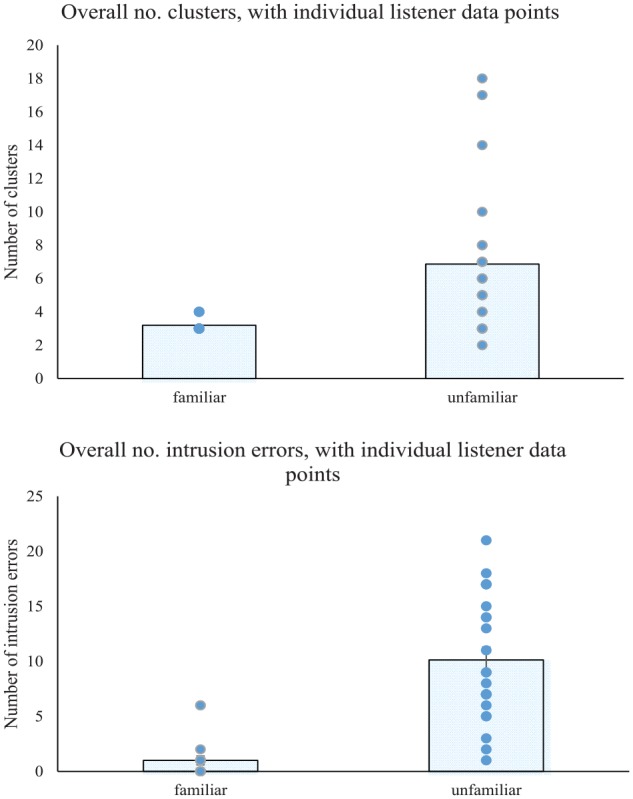
Top panel: number of perceived identity clusters for familiar and unfamiliar listeners. Bars show the means across listeners, and dots show the participant data points, with ties depicted by a single dot. Bottom panel: number of intrusion errors for familiar and unfamiliar listeners. Bars show the means across listeners, and dots show the participant data points, with ties depicted by a single dot.

The perceived number of identity clusters was determined by examination of the spatial arrangement of loudspeaker icons on the PowerPoint slide at the end of the “Mr Tickle” trial. This represented the number of speakers that each participant thought was present across the 52 Mr Tickle clips and, in essence, this reflected the ability of the listener to tell different clips of the same speaker together. In this regard, the familiar group indicated between 3 and 4 identities, with a mean of 3.20 identities (*SD* *=* .41), and a modal value of 3 identities indicated by 16/20 participants. In contrast, the unfamiliar group indicated between 2 and 18 identities, with a mean of 6.87 identities (*SD* = 4.28) and a modal value of 3 identities indicated by 4/23 participants^1^ (see Figure 2 and Table 1).

**Table 1.** Mean, median, range, and mode of the number of identity clusters and intrusion errors in the voice sorting task, together with self-rated confidence (out of 7).

	Familiar listeners	Unfamiliar listeners
Identity clusters		
*M* (*SD*)	3.20 (0.41)	6.87 (4.28)
Median	3	6
Range	3–4	2–18
Mode	3	3
Intrusion errors		
*M* (*SD*)	1.00 (1.81)	10.13 (5.64)
Median	0	9
Range	0–6	1–21
Mode	0	7
Confidence (/7)		
*M* (*SD*)	5.70 (1.13)	3.61 (1.37)
Median	6	3
Range	4–7	2–6
Mode	6	3

Two Bonferroni-corrected one-sample Wilcoxon signed-rank tests revealed that the familiar group produced a solution which did not differ from the truth (three clusters) (*W* = 2.00, *p* > .025), whereas the unfamiliar group produced a solution which deviated significantly from the truth (*W* = 3.75, *p* < .001). Moreover, direct comparison using a Mann–Whitney *U* test showed that the familiar and unfamiliar groups differed significantly from one another in the perceived number of identity clusters (*U* = 4.07, *p* = .001). As such, the results indicated that familiarity with the speakers improved the ability to tell different clips of the same speaker together. These results thus confirmed the predictions based on results of the face sorting task by Andrews et al. (2015) and Jenkins et al. (2011) and the voice sorting task by Lavan et al. (2018; Lavan, Burston, et al., 2019).

### Number of intrusion errors

The number of intrusion errors was determined in the same way as Jenkins et al. (2011) and reflected the purity of the identity clusters in a participant’s final solution. An intrusion error was defined as the presence of a clip belonging to one speaker within a cluster that predominantly contained clips of another speaker. In essence, this reflected the ability of the listener to tell similar clips from different speakers apart, with a higher number of intrusion errors indicating a poorer ability. Each intruder clip was counted once whether they reflected the same “intruder” or different “intruders.” For instance, a cluster of clips belonging to Speaker A, with an intruder clip from Speaker B and two intruder clips from Speaker C would be classed as showing three intrusion errors. In clusters where there was no majority speaker, such as when one clip from Speaker A was paired with one clip from Speaker B, then the cluster was arbitrarily assigned to one identity, and the number of “intruder” clips counted relative to that identity (see Figure 2 and Table 1).

Examination of the number of intrusion errors across the familiar and unfamiliar participant groups revealed a mean of 1.00 errors (*SD* = 1.81) in the familiar group and a mean of 10.13 errors (*SD* = 5.64) in the unfamiliar group. More specifically, 11 of the 20 familiar participants reached a perfect solution involving three pure clusters and no intrusion errors, and another six participants made only one intrusion error out of the entire set of 52 clips. In contrast, none of the unfamiliar participants reached a pure three-cluster solution, and instead the unfamiliar group showed a modal value of 7 errors made by 4/23 participants.

Comparison by means of Mann–Whitney *U* test indicated that the two groups differed significantly in the number of intrusion errors (*U* = 5.28, *p* < .001), suggesting that familiarity with the speakers improved the ability to tell voices apart rather than confuse them into mixed-identity clusters. This pattern of errors contrasted with that in the face sorting task (Andrews et al., 2015; Jenkins et al., 2011) and the voice sorting task (Lavan et al., 2018; Lavan, Burston, et al., 2019) where the number of intrusion errors was very low for both listener groups. This perhaps reflected the relative difficulty of the face discrimination and voice discrimination tasks per se, but is a point that is considered further in the Discussion.

### Within-identity clustering: telling together

**Figure 3.**
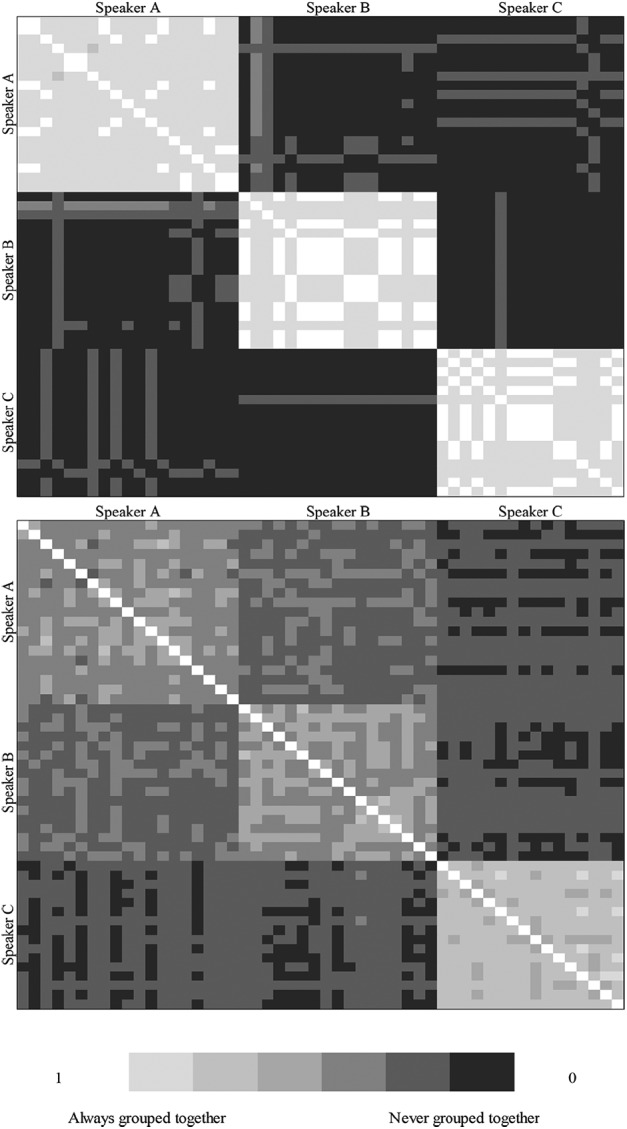
Top Panel: Matrices of averaged listeners’ responses for familiar listeners. Bottom Panel: Matrices of averaged listeners’ responses for unfamiliar listeners. The matrices depict the probability with which each clip for the three speakers is paired with other clips from the same speaker (regions hugging the diagonal) and other clips from different speakers (three regions towards the top right and bottom left). A light value indicates a high probability of being clustered together and is thus expected in within-identity regions. A dark value indicates a low probability of being clustered together and is thus expected in cross-identity regions.

To provide comparability with the results of Lavan et al. (2018; Lavan, Burston, et al., 2019), individual participant response matrices were generated, representing the grouping of each of the 52 clips with each of the other 51 clips. Replicating the approach taken by Lavan et al., a coding of 1 indicated that two clips were sorted into the same cluster and a coding of 0 indicated that they were not. The resultant matrix for each individual thus illustrated the ability to tell clips of each identity together (in within-identity regions of the matrix) and the ability to tell them apart (in cross-identity regions of the matrix). Figure 3 shows the group averaged matrices, shaded for ease of inspection. A light-coloured cell is indicative of two clips being grouped together and thus is expected in within-identity regions. Conversely, a dark-coloured cell is indicative of two clips being grouped apart and thus is expected in cross-identity regions. By definition, the matrices are symmetrical along the diagonal, and the diagonal represents the (constant) grouping of each clip with itself and is thus meaningless.

Analysis was conducted on the overall within-identity score (combining the within-identity clusters across all three speakers). This revealed significantly better performance among familiar listeners than unfamiliar listeners (*U* = 5.29, *p* < .001). Moreover, this benefit held when each of the three speakers was taken separately (Speaker A: *U* = 5.09, *p* < .001; Speaker B: *U* = 4.72, *p* < .001; Speaker C: *U* = 3.40, *p* = .001). Thus, familiarity enabled better performance when mapping two clips of the same speaker together.

**Figure 4.**
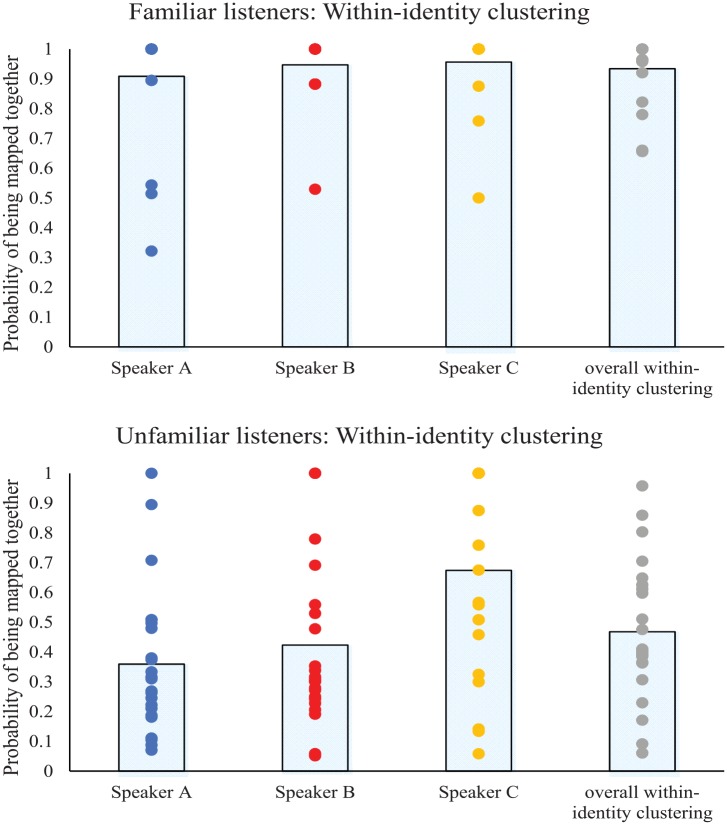
Within-identity clustering for each speaker, and for all three speakers overall. Bars indicate the mean probability of being mapped together, whereas dots indicate individual participant scores (ties are represented by a single dot). A high score indicates a high probability of clips being mapped together. Top Panel: Results for familiar listeners. Bottom Panel: Results for unfamiliar listeners where a significant difference emerged in within-identity clustering across the three speakers.

With data broken down for the three speakers, it was possible to determine whether the ability to map clips together was uniform across the three identities. A Friedman Two-Way Analysis of Variance (ANOVA) by Ranks was conducted for the familiar listeners and for the unfamiliar listeners taken separately. This revealed no significant difference across the three speakers when the listeners were familiar with them, *FM*_(2)_ = 1.19, *p* = .552, suggesting that all three speakers could be clustered together with equal ease. In contrast, a significant difference emerged across speakers when the listeners were unfamiliar with them, *FM*_(2)_ = 20.21, *p* < .001. Nonparametric and Bonferroni-corrected pairwise comparisons indicated that Speaker C was clustered together better than both Speaker A (*W* = 3.88, *p* < .001) and Speaker B (*W* = 3.10, *p* = .002), and that Speaker A and Speaker B did not differ from one another (*W* = 1.95, *p* = .052). Thus, although Speaker C was better clustered together when familiar than when unfamiliar (above), it was nevertheless better clustered together by unfamiliar listeners compared with the other two speakers (see Figure 4 and Table 2).

**Table 2.** Mean (with standard deviation), median, and modal values for within-identity clustering for each speaker, and averaged across all speakers.

	Speaker A	Speaker B	Speaker C	Within-identity
Familiar listeners				
*M* (*SD*)	0.91 (0.20)	0.95 (0.11)	0.96 (0.12)	0.93 (0.11)
Median	1	1	1	1
Mode	1	1	1	1
Unfamiliar listeners				
*M* (*SD*)	0.36 (0.24)	0.42 (0.29)	0.67 (0.32)	0.47 (0.23)
Median	0.31	0.31	0.76	0.41
Mode	0.51	1	1	0.39

Performance is shown for familiar and unfamiliar listener groups. A high score indicates a high probability of clips being mapped together.

### Cross-identity confusion: telling apart

While the analysis above concentrated on clustering of clips with others of the same identity, the matrices also revealed the tendency to cluster clips with others of different identities. These cross-identity confusions represented a failure to tell different speakers apart and were revealed by high scores (light squares) within the cross-identity regions of the matrix.

**Figure 5.**
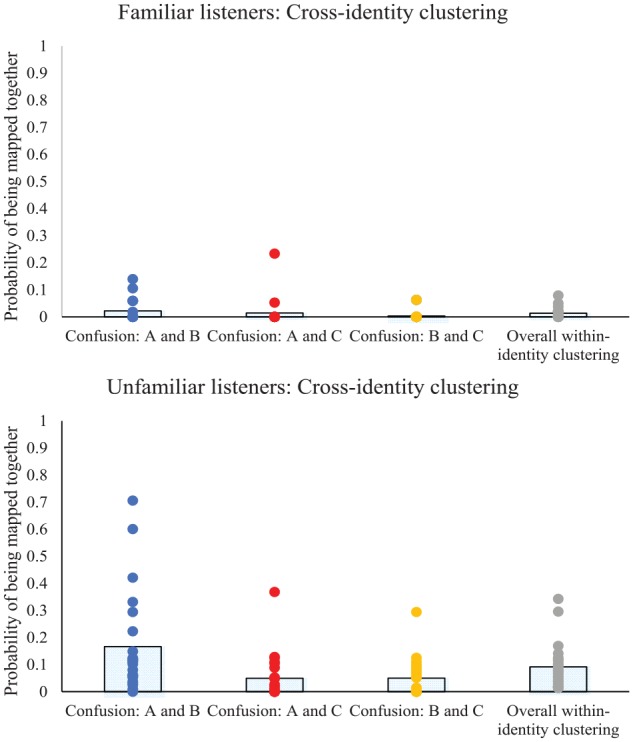
Cross-identity clustering for each speaker, and for all three speakers overall. Bars indicate the mean probability of being mapped together, whereas dots indicate individual participant scores (ties are represented by a single dot). A high score indicates a high probability of clips being inappropriately mapped together. Top Panel: Results for familiar listeners. Bottom Panel: Results for unfamiliar listeners where a significant difference in confusability emerged across the three speakers.

Analysis was conducted on the overall cross-identity scores (combining cross-identity clusters across all three speakers). This revealed significantly better performance for familiar listeners than for unfamiliar listeners in the form of lower scores for cross-identity clusters (*U* = 4.45, *p* < .001). As above, this benefit held when confusion of each speaker with each other speaker was analysed in turn (confusion of Speakers A and B: *U* = 4.31, *p* < .001; confusion of Speakers A and C: *U* = 3.49, *p* < .001; confusion of Speakers B and C: *U* = 3.71, *p* < .001). Thus, familiarity enabled a better performance when mapping two different speakers apart (see Figure 5 and Table 3). As with the number of intruders considered previously, the pattern here deviated from that with faces (Andrews et al., 2015; Jenkins et al., 2011) and from that with voices in previous studies (Lavan et al., 2018; Lavan, Burston, et al., 2019).

**Table 3.** Mean (with standard deviation), median, and modal values for cross-identity clustering for each speaker, and averaged across all speakers.

	Speakers A-B	Speakers A-C	Speakers B-C	Cross-identity
Familiar listeners				
*M* (*SD*)	0.02 (0.04)	0.01 (0.05)	0.00 (0.01)	0.01 (0.02)
Median	0	0	0	0
Mode	0	0	0	0
Unfamiliar listeners				
*M* (*SD*)	0.17 (0.19)	0.05 (0.08)	0.05 (0.07)	0.09 (0.08)
Median	0.11	0.01	0.01	0.07
Mode	0.11	0	0	0.02

Performance is shown for familiar and unfamiliar listener groups. A high score indicates a high probability of clips being inappropriately mapped together.

To determine whether any of the speakers was any more confusable than the others, analysis of the confusion between speakers was examined for each confusion pair in turn, within each of the listener groups. A Friedman Two-Way ANOVA by Ranks was again used. This revealed no significant difference in confusability for the three pairs of identities when listeners were familiar with the speakers, *FM*_(2)_ = 4.67, *p* = .097. Indeed, the probability of speaker confusion for each of the pairs suggested that confusion was relatively infrequent. In contrast, analysis of cross-identity confusions among unfamiliar listeners revealed a significant difference in the confusability of the three pairs of identities, *FM*_(2)_ = 23.88, *p* < .001. Nonparametric and Bonferroni-corrected pairwise comparisons revealed that confusion of either Speaker A or B with Speaker C was relatively rare, and was significantly less frequent than confusion of Speakers A and B (AB vs. AC: *W* = 3.36, *p* < .001; AB vs. BC: *W* = 3.82, *p* < .001; AC vs. BC: *W* = .196, *p* = .845). Thus, Speaker C was mistakenly clustered less often when listeners were familiar with the speaker than when unfamiliar, but nevertheless, Speaker C was mistakenly clustered less often than Speakers A and B even when listeners were unfamiliar with all voices.

### Misidentification index and speaker confusability

The final measure of the accuracy of telling together and telling apart was the misidentification index as used by Lavan, Burston, et al. (2019). This was calculated by subtracting the probability of a mistaken clustering from the probability of an accurate clustering for each voice clip (*P*(within-identity score) – *P*(cross-identity score)). Scores varied between 0 and 1, with a score of 1 indicating perfect clustering of a clip with other clips from the same speaker, and never with other clips from different speakers.

**Figure 6.**
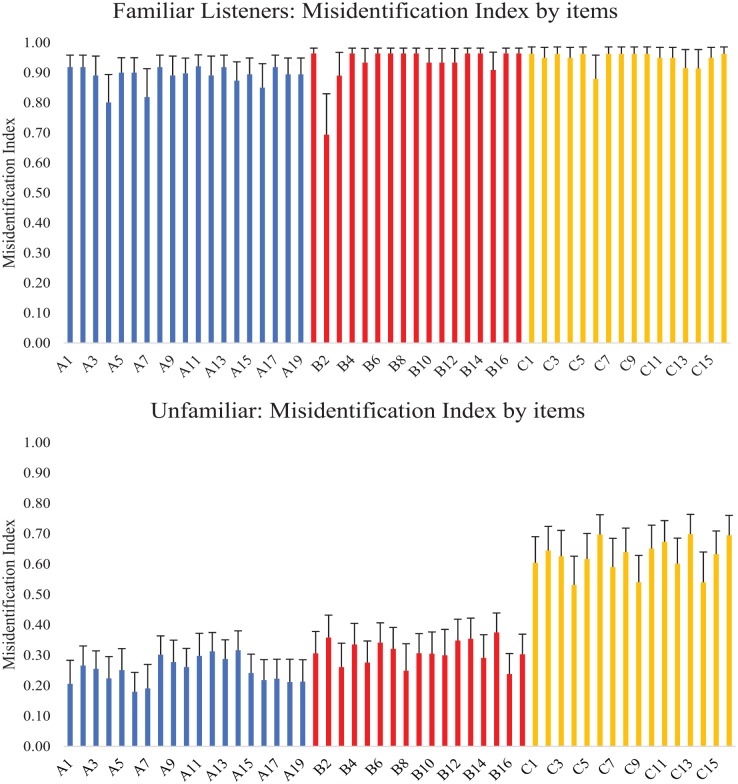
Misidentification index (*P*(within-identity match) – *P*(cross-identity match)) for each item. A high score indicated better clustering with clips of the same identity than with clips from different identities. Top Panel: Results averaged across familiar listeners. Bottom Panel: Results averaged across unfamiliar listeners.

The misidentification index was calculated on a participant by participant basis for each and every clip (see Figure 6). When averaged across the clips associated with each speaker, this yielded a single score representing the misidentification index for that speaker. Comparison across listener groups by means of three Mann–Whitney *U* tests revealed better performance for familiar than for unfamiliar listeners for each of the speakers (Speaker A: *U* = 5.22, *p* < .001; Speaker B: *U* = 5.13, *p* < .001; Speaker C: *U* = 4.07, *p* < .001), suggesting that familiar listeners were more able both when telling together clips from the same speaker *and* when telling apart clips from different speakers.

As above, a Friedman Two-Way ANOVA by Ranks was used to see whether the average misidentification index across sets of clips differed for the three speakers. For familiar listeners, no significant difference was evident, *FW*_(2)_ = 1.56, *p* = .459, suggesting that all three speakers could be mapped together and mapped apart with equal ease. In contrast, and as with the previous results, unfamiliar listeners showed a significant difference across the three speakers, *FW*_(2)_ = 22.52, *p* < .001. Nonparametric, Bonferroni-corrected pairwise comparisons suggested that the misidentification index showed significantly better performance for Speaker C compared with Speaker A (A vs. C: *W* = 4.11, *p* < .001) and compared with Speaker B (B vs. C: *W* = 3.38, *p* < .001). However, Speakers A and B did not differ (A vs. B: *W* = 1.74, *p* = .078).

### Analysis of speaker characteristics

Analysis of the speaker characteristics associated with each speaker provided some evidence in support of the differences in speaker confusability noted above. PRAAT (Version 6.0.43) was used to extract a number of vocal characteristics (see Table 4). These focussed on measures associated with fundamental frequency (F0), and formant characteristics, given their prominence within the literature (see Baumann & Belin, 2010; Latinus & Belin, 2011). Characteristics were extracted from manually defined voiced segments of each speech clip, using settings appropriate for normal adult speakers (pitch range = 75–300 Hz, intensity range = 50–100 dB).

**Table 4.** Mean vocal characteristics across the clips within each speaker set (with standard deviation).

	Speaker A		Speaker B		Speaker C	
	*M*	(*SD*)	*M*	(*SD*)	*M*	(*SD*)
F0 Mean	204.17	(12.3)	201.90	(12.3)	193.93	(14.6)
F0 Standard deviation	39.15	(9.5)	37.78	(7.8)	40.62	(9.4)
F0 Minimum	104.05	(34.6)	125.97	(25.6)	96.20	(23.8)
F0 Range	180.24	(42.6)	168.89	(35.8)	183.30	(43.1)
Harmonics-to-noise ratio	9.55	(1.2)	8.76	(0.9)	8.42	(2.4)
F1	598.70	(58.0)	614.07	(56.2)	650.48	(80.9)
F2	1,986.10	(193.5)	1,815.62	(93.4)	1,734.38	(135.3)
F3	3,027.70	(79.8)	2,993.30	(100.9)	2,929.64	(67.4)
F4	4,084.66	(119.9)	4,074.01	(111.0)	3,887.69	(150.1)
Formant dispersion	1,161.99	(46.1)	1,153.31	(39.0)	1,079.07	(48.9)

**Figure 7.**
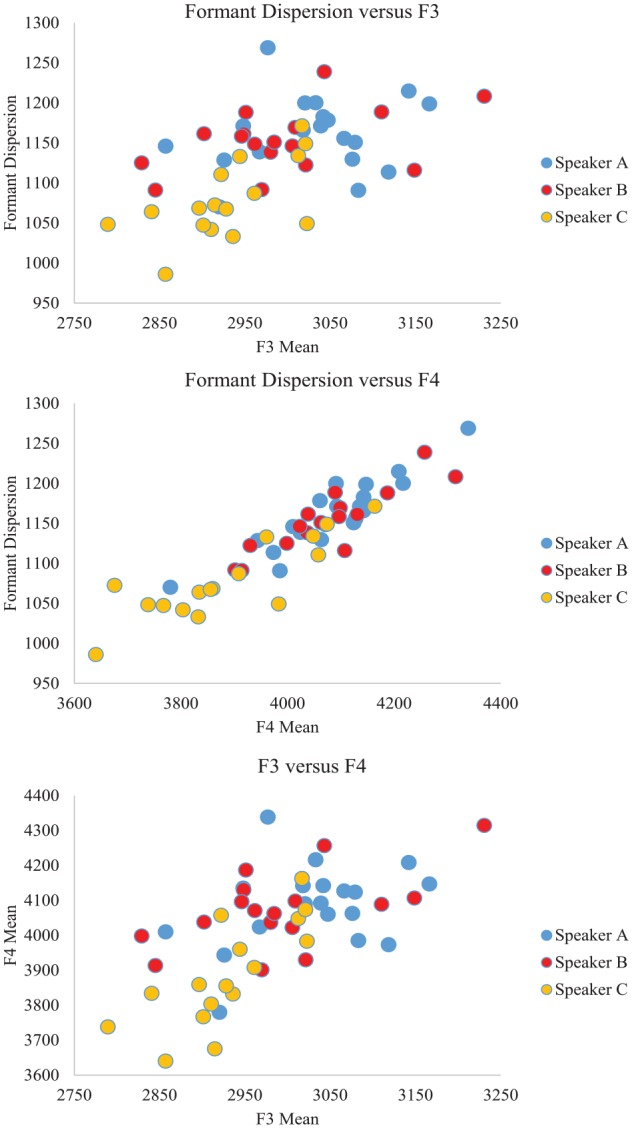
Scatterplot of the clips of each speaker set, organised according to the vocal characteristics that differentiated best between speakers (Formant Dispersion, F3, and F4). Within each plot, the dots associated with Speaker C tended to be differentiated from those associated with Speakers A and B.

A series of between-items nonparametric Kruskal–Wallis ANOVAs were conducted to determine whether the three speakers differed on any of the extracted vocal characteristics. Correcting for the number of tests performed by adopting an alpha level of .005, these analyses revealed significant speaker differences in four of the 10 measures. Bonferroni-corrected pairwise comparisons were conducted to establish the pattern of differences across the three speakers. These typically revealed a difference between Speaker C and one or both of the other speakers (see Table 5 and Figure 7). This supported the findings above that Speaker C was least often confused based on the misidentification index (see Figure 6) because Speaker C stood apart from the other two speakers. In contrast, Speakers A and B were seen to differ on only one of the vocal characteristics (second formant characteristic, F2) supporting the observation of their high rate of confusability.

**Table 5.** Exploration of vocal characteristics across speakers, together with Standardised Test Statistics for Bonferroni-corrected pairwise comparisons.

	Overall difference	Kruskal–Wallis result	Speaker A vs. B	Speaker A vs. C	Speaker B vs. C
F0 Mean	*ns*	KW_(2)_ = 4.16, *p* = .125			
F0 Standard deviation	*ns*	KW_(2)_ = 1.23, *p* = .542			
F0 Minimum	*ns*	KW_(2)_ = 9.13, *p* = .010			
F0 Range	*ns*	KW_(2)_ = 2.16, *p* = .339			
Harmonics-to-noise ratio	*ns*	KW_(2)_ = 6.94, *p* = .031			
F1	*ns*	KW_(2)_ = 7.22, *p* = .027			
F2	sig	KW_(2)_ = 17.79, *p* < .001	*z* = –2.87, *p* = .012	*z* = 4.07, *p* < .001	*z* = 1.22, *p* = .67
F3	sig	KW_(2)_ = 12.21, *p* = .002	*z* = –1.42, *p* = .466	*z* = 3.49, *p* = .001	*z* = 2.03, *p* = .126
F4	sig	KW_(2)_ = 15.48, *p* < .001	*z* = –0.47, *p* = 1.00	*z* = 3.68, *p* = .001	*z* = 3.13, *p* = .005
Formant dispersion	sig	KW_(2)_ = 19.64, *p* < .001	*z* = –0.51, *p* = 1.00	*z* = 4.14, *p* < .001	*z* = 3.54, *p* = .001

Surprisingly, there were no overall differences in any of the four measures connected to fundamental frequency (perhaps because all speakers were female), or in harmonics-to-noise ratio (HNR) and first formant characteristic (F1). As such, this analysis offered some insight into the vocal characteristics that contributed to the observed pattern of confusability while acknowledging that additional characteristics not captured here may also contribute to performance.

### A consideration of particular items

Graphical examination of the misidentification index per clip averaged for each listener group (Figure 6) suggested that some clips were harder to tell together and tell apart than others, as indicated by lower misidentification index scores. Visual analysis highlighted two clips for Speaker A (A4, A7), one clip for Speaker B (B2), and three clips for Speaker C (C6, C13, C14) which stood out from their respective sets. This was confirmed by their identification as outliers (according to the standard of 1.5 x interquartile range above the Q3 value or below the Q1 value).

Somewhat surprisingly, an exploratory analysis of the acoustic properties associated with these clips suggested that they did not stand out as outliers from their respective identity sets on any of the extracted vocal measures. It was notable that A4 was particularly short (1.02 s of speech relative to a mean speech length of 2.42 s). The short duration may have made it difficult for the listener to extract key vocal characteristics, with a consequential rise in confusability. In addition, B2 had the one of the highest maximum pitch values in that speaker set, with an initial and sustained high pitch that better resembled Speaker A. Indeed, Speaker A (the first author) identified herself in this clip on first listening. Nevertheless, the current set of metrics make it difficult to attribute this confusion to a particular measurable characteristic. In this sense, it is clear that voice clips vary in multiple dimensions and this richness is undoubtedly not fully captured by the metrics selected here. Much more work would be required to untangle the speaker characteristics contributing to confusability within identity sets. However, the analysis above has presented some helpful contenders when examining vocal characteristics at the level of the speaker if not at the level of the individual clip.

### Correlations between measures

One interesting question concerned the extent to which the capacity to tell different clips of the same speaker together was associated with the capacity to tell similar clips of different speakers apart. To this end, a Spearman’s bivariate correlation was computed between the number of identity clusters, and the number of intrusion errors for the familiar group and the unfamiliar group separately. This revealed a strong and significant correlation between the two measures when familiar with the speakers, *r*_(20)_ = .63, *p* = .003, but not when unfamiliar with the speakers, *r*_(23)_ = –.11, *p* = .63. In addition, when the more nuanced averaged matrix scores were considered, the ability to tell voices together in within-identity regions was strongly correlated with the ability to tell voices apart in cross-identity regions when familiar with the speakers, *r*_(20)_ = .943, *p* < .001, but not when unfamiliar with the speakers, *r*_(23)_ = .212, *p* = .333. This suggested that familiar listeners were able to both tell voices together and tell voices apart, whereas the unfamiliar listeners showed no association between these two capabilities.

### Self-rated confidence

Finally, the current design permitted examination of participants’ self-rated confidence in the final solution, and this provided a metacognitive measure of performance alongside the behavioural measures above (see Table 1). Comparison by means of a Mann–Whitney *U* test showed a clear and significant difference (*U* = 4.18, *p* < .001) such that participants who were familiar with the speakers expressed far higher confidence in their final solution (*M* = 5.70, *SD* = 1.13) than participants who were unfamiliar with the speakers (*M* = 3.61, *SD* = 1.37).

## Discussion

The current study explored the performance of familiar and unfamiliar listeners when processing vocal identity. The use of a voice sorting task with familiar and unfamiliar listeners allowed performance to be evaluated using a single set of stimuli and a common task, and this represented an improvement over previous approaches. With this cleaner methodology, the current results highlighted several notable findings.

First, familiar listeners were significantly more able than unfamiliar listeners to tell different instances of the same speaker together, as indicated by both the number of resultant identity clusters following sorting, and the within-identity clustering scores. This outcome met with expectations following the use of the sorting task with faces (Andrews et al., 2015; Jenkins et al., 2011) and the use of the sorting task with voices (Lavan et al., 2018; Lavan, Burston, et al., 2019). Furthermore, it cements the conclusion that familiarity with an identity helps the perceiver to map different instances of that identity together despite inherent variability between one instance and the next.

Second, familiar listeners performed significantly better than unfamiliar listeners when the misidentification index was considered. Given the advantage in within-identity clustering discussed above, a benefit of familiarity when considering this overarching measure was perhaps to be expected. Added to this, familiar listeners were more confident in their sorting ability than unfamiliar listeners suggesting a benefit at a metacognitive level as well as at a behavioural level.

As an extension to previous work, the current study also enabled an examination of the variation in ability to sort both across speakers and across individual clips in each speaker set. In this regard, although familiar listeners could tell together and could tell apart instances of all three speakers, and were better than the unfamiliar listeners for all three, the unfamiliar listeners found some speakers easier to sort than others. Analysis of the vocal characteristics suggested some differentiation of the speakers on characteristics related to formant characteristics. In fact, one voice stood apart from the other two and may be regarded as distinctive. As a result, this distinctive speaker was easier to tell together (higher within-identity clustering) and was easier to tell apart from the other speakers (lower cross-identity clustering). Interestingly, the relative ease of sorting associated with this distinctive speaker was not sufficient to remove a benefit of familiarity with the speaker’s voice here. Somewhat surprisingly, the analysis of vocal characteristics did not help to elucidate why some clips within each speaker set caused more problems than others. Nevertheless, the analysis suggested how vocal characteristics may be useful in spotting distinctiveness and thus in spotting ease of performance in a sorting task. The benefit of knowing this is that it serves as a reminder that items effects can represent an important consideration especially in studies which use few speakers as items.

In one respect, the results of the present sorting task were, however, surprising. Specifically, familiar listeners were significantly more able to tell similar instances of two different speakers apart, as indicated by fewer intrusion errors following sorting, and lower cross-identity clustering scores. This better performance with familiarity aligns well with the results discussed above. Nevertheless, this particular benefit when telling voices apart was not predicted given that all previous uses of the sorting task had suggested a low incidence of mixed clusters for familiar and unfamiliar participants alike, both when processing faces (Andrews et al., 2015; Jenkins et al., 2011) and voices (Lavan et al., 2018; Lavan, Burston, et al., 2019).

This difference in results may be explained by considering the differences in stimuli across studies. For instance, a difference between the results with faces and those here with voices may be explained by the fact that face processing is a somewhat easier task than voice processing (see Gainotti, 2011; Hanley et al., 1998). Consequently, when faces were considered, unfamiliar perceivers were able to complete one of the two aspects tested by the sorting paradigm (they could tell faces apart as effectively as familiar perceivers). In contrast, and given the relative difficulty of the voice processing task, unfamiliar listeners here struggled with both aspects tested by the sorting paradigm (both telling voices apart and telling voices together).

This said, the current voice sorting task provided a different pattern of performance compared with Lavan et al.’s (2018; Lavan, Burston, et al., 2019) voice sorting tasks and this warrants closer inspection. In this regard, several differences existed between the current and previous voice sorting studies. First, the current study used British listeners and British speakers which avoided any difficulties associated with processing an unfamiliar accent (see Stevenage, Clarke, & McNeill, 2012). By comparison, the studies used by Lavan et al. (2018; Lavan, Burston, et al., 2019) used speakers with American English accents and may have introduced an other-accent effect when testing a British University participant pool. The possibility of other-accent effects thus cannot be ruled out. This being the case though, it is difficult to see why the unfamiliar listeners used by Lavan et al. performed well and were equivalent to the familiar listeners when telling voices apart despite the possibility of an other-accent effect (while those tested here were worse than the familiar listeners). This suggests that although it may be best to avoid the introduction of an other-accent affect, it may not account for the difference in results across studies.

A second difference between the present study and those of Lavan et al. (2018; Lavan, Burston, et al., 2019) relates to the use of three speakers with unequal set sizes in the current study as opposed to two speakers with equal set sizes in previous studies. This is perhaps a trivial point but it may carry a perceptual consequence for the participants in that the use of three speakers will have resulted in a voice set displaying greater vocal variability than that associated with just two speakers. Familiar listeners could resolve this variability well. However, this variability may have contributed both to the perception of more identities (more clusters) but also to greater confusion between identities (more intrusion errors or cross-identity grouping) among unfamiliar listeners than is evident in previous voice sorting tasks. As such, the use of three identities within the current sorting task arguably may have provided a more realistic test of voice sorting ability which enabled confusion errors to emerge in the unfamiliar listeners.

A third difference between the present study and those of Lavan et al. (2018; Lavan, Burston, et al., 2019) is the use of scripted speech (here) versus spontaneous-yet-acted speech in the previous studies. In this regard, it is possible that the use of scripted speech here resulted in clips that were more uniform across speakers, with the result that telling voices apart was more difficult in the present study, especially for unfamiliar listeners. This may have removed any ceiling effects present in Lavan et al.’s studies, enabling a difference between familiar and unfamiliar listeners to emerge in cross-identity confusions as well as in the number of perceived identities. Without any vocal metrics associated with Lavan, Burston, et al.’s (2019) voice clips, the possibility of a difference in uniformity across scripted and spontaneous clips is difficult to evaluate. In contrast, the vocal metrics reported by Lavan et al. (2018) and the vocal metrics reported here did not readily account for the pattern of performance when sorting individual speaker clips. Nevertheless, variability of the clips used across studies certainly warrants further attention, with the current data suggesting that differences at the speaker level may be indicative of differences in sorting ability.

Finally, the current study used personally familiar voices as stimuli rather than publicly familiar (celebrity voices) as used by Lavan et al. (2018; Lavan, Burston, et al., 2019). In the face domain, the basis for familiarity has been the focus of some discussion, with several authors questioning the equivalence of personally and publicly familiar stimuli (see Carbon, 2008; Ramon, Caharel, & Rossion, 2011; Tong & Nakayama, 1999; Wiese et al., 2018). In particular, a concern centred on the possibility that celebrity face processing may reflect item-specific processing relative to a particular stored iconic image rather than processing at the level of the identity itself. Indeed, celebrity recognition was shown to be relatively poor when presented with slightly modified or unfamiliar versions of celebrity faces (Carbon, 2008). In contrast, the processing of a personally familiar individual may rely on a stored mental representation that is richer, more representative, or what Tong and Nakayama (1999) referred to as more robust. In the context of the current study, there is no reason to suppose that the distinction between personally and publicly familiar stimuli cannot be generalised from the face domain to the voice domain. In this regard, the existence of a stronger mental representation for personally familiar voices here may have contributed to the processing differences across studies.

### So what differs between the processing of familiar and unfamiliar listeners?

A discussion of the difference in stored representation across different levels of familiarity starts to enable a consideration of what may differ between the voice processing of an unfamiliar listener and the voice processing of a familiar listener. One appealing representational framework draws on the concept of a similarity space (see Leopold, O’Toole, Vetter, & Blanz, 2001; Valentine, 1991). When presented with an unfamiliar stimulus, a perceiver may locate that stimulus within the similarity space based on a superficial analysis of available characteristics, and this may support a temporary ability in a matching task or a discrimination task. However, true recognition arguably depends on the existence of a pre-existing and stored mental representation within the similarity space which is triggered upon presentation of a familiar and recognisable individual.

In the voice domain, the concept of a similarity space has been explored, and Baumann and Belin (2010) have identified two cardinal dimensions along which voices may be differentiated—fundamental frequency and formant characteristics. At its simplest level, each voice identity may thus be located as a point within this two-dimensional voice space, with this point representing an average or a prototype extracted from all experienced instances (see Andics et al., 2010, for discussion of prototype extraction in the voice domain). The success of recognition depends upon the proximity of an instance to its stored prototype rather than to its next nearest neighbour. A point-based representation within similarity space is thus good at accounting for the ability to tell different individuals apart.

This said, it is plausible to consider that, with increasing familiarity comes a refinement of the stored mental representation for a known individual (Lavan, Burton, Scott & McGettigan, 2019). Indeed, Tong and Nakayama linked the formation of robust representations to the development of extensive experience or familiarity, with the most robust representations existing for the most familiar stimuli one experiences (such as self, partner or family members). Our contention given the results of the present study is that increasing familiarity may enable the development of a representation which captures both the variability within individuals as well as the separation between individuals. This would enable the perceiver to be able to map different instances of the same individual together as well as to map different individuals apart.

At a theoretical level, one way to conceptualise this refinement of the stored representation with increasing familiarity is to consider a shift from a representation as a *point* in similarity space to a representation as a *region* within similarity space (see Lewis & Johnston’s, 1999, discussion of Voronoi Cells). Although a point-based representation may enable separation of different identities and may be laid down first, a region-based representation captures the variability of different instances of that identity, and reflects what Vernon (1952, cited in Bruce, 1994, p. 8) described as the “possible and permissible variations” within an identity. This concept of a representational region is far more than a mechanism to enable a perceiver to develop a tolerance band to cope with noisy or suboptimal presentations, as it may have historically been viewed (see Valentine, 1991). Instead, it is a representation of the meaningful variability that an individual may display across different moments in time. Logically, an appreciation of the variability of an individual may take time, experience, and familiarity to develop, but the consequence of this representational refinement is that a perceiver becomes able to tell different instances of the same person together, as well as being able to tell similar instances of two different people apart.

A consideration of an identity region provides a useful way of accounting for the current results. However, this sort of representational framework can also be extended to incorporate Burton’s (2013) recent thinking on variability as a cue to identity in and of itself. Burton considered that, rather than variability merely reflecting noise, the pattern of variability that an individual displays may be a characteristic of that individual’s identity in and of itself. In this regard, it is quite possible that one identity may display more or less variability than another, and this may be a meaningful element for a mental representation to capture. This can be accommodated into a region-based view of representations by assuming regions of different sizes for different identities. Accordingly, the success of recognition now depends upon the overlap between these identity regions and this itself depends upon both the proximity of the region to its nearest neighbour (to tell them apart), and the variability within the identity, or size of the region (to tell the instances of one identity together). In the voice domain, one study has recently begun to quantify variability across different instances within an identity using everyday speech sessions (Kreiman, Park, Keating, & Alwan, 2015). However, it would be interesting to explore whether there is any link between intra-speaker variability, vocal distinctiveness, and performance in a sorting or discrimination task along the lines discussed.

### Conclusions and final thoughts

In summary, the present study has used a voice sorting task to show a difference in vocal identity processing across personally familiar listeners and unfamiliar listeners. Specifically, personally familiar listeners were better able to tell voices together as shown through the creation of fewer identity clusters and higher within-identity clustering scores. In contrast to previous studies, personally familiar listeners were also better able to tell voices apart, as shown through fewer intrusion errors and lower cross-identity clustering scores. Interestingly, familiarity also led to higher metacognitive evaluations of performance. Although the performance of unfamiliar listeners was significantly worse than that of familiar listeners, it was notable that the performance of unfamiliar listeners was influenced by the voices themselves, with some voices being easier to sort than others. In accounting for these results, a theoretical framework has been discussed which links familiarity with the development of a robust representation capable of capturing both within-identity variation as well as between-identity separation. This may best be reflected by a region rather than a point within a representational space, and a shift to this type of thinking may enable new questions to be asked and answered.

In line with the thinking proposed here, a final observation is to note a similarity between the themes we summarise above, and those discussed with the categorical perception literature (see Bornstein, 1987; Liberman, Harris, Hoffman, & Griffith, 1957). Indeed, the categorical perception concepts of within-category compression and between-category separation map nicely onto the concepts of telling together and telling apart, respectively. An interesting literature has tracked the emergence of within-category compression and between-category separation as a consequence of category learning (see Goldstone, 1994; Livingston, Andrews, & Harnad, 1998; Schyns & Rodet, 1997), where categories can refer to different identities (see Beale & Keil, 1995; Stevenage, 1998). In this regard, the literatures relating to familiarity effects in (face and) voice processing may usefully be aligned to the very established literature on categorical perception, with the potential to take both our methodology and our theoretical understanding forward.

## Supplemental Material

Supplementary_Evidence_Correlational_Study_R2 – Supplemental material for Sorting through the impact of familiarity when processing vocal identity: Results from a voice sorting task Click here for additional data file. Supplemental material, Supplementary_Evidence_Correlational_Study_R2 for Sorting through the impact of familiarity when processing vocal identity: Results from a voice sorting task by Sarah V Stevenage, Ashley E Symons, Abi Fletcher and Chantelle Coen in Quarterly Journal of Experimental Psychology

## Notes

1. Although four participants in the unfamiliar group apparently reached the correct solution of 3 identity clusters, their clusters were nevertheless mixed in terms of the speakers contributing to them. Thus, although correct in their number of speakers, they could not tell the speakers together, or apart from one another.
