# Supplementary material for: Sorting through the impact of familiarity when processing vocal identity: Results from a voice sorting task
Source: Q J Exp Psychol (Hove). 2019 Nov 22;73(4):519–36. doi: 10.1177/1747021819888064 (PMC7074657; doi:10.1177/1747021819888064)
Supplement: Supplementary_Evidence_Correlational_Study_R2 – Supplemental material for Sorting through the impact of familiarity when processing vocal identity: Results from a voice sorting task [file Supplementary_Evidence_Correlational_Study_R2.docx]

Consideration of a Distinction between Familiar and Unfamiliar Vocal Identity Process: Analysis across Tasks

Consideration of a distinction between familiar and unfamiliar identity processing has been extended from the face domain (Megreya & Burton, 2006) to the voice domain. This was first highlighted by van Lancker and Kreiman (1987) who showed a dissociation between familiar and unfamiliar voice processing through the examination of capabilities within clinical groups. More recently, however, this distinction has been the focus of several reviews (Gainotti, 2011; Stevenage, 2017) which have highlighted a distinction at a neurophysiological level (Warren, Scott, Price & Griffiths, 2006), as well as at the behavioural level in neurotypical and single case study clinical accounts (see review by Stevenage, 2017). On the basis of this literature, a distinction between familiar and unfamiliar voice processing appears to be warranted, and a correlation between performance when processing familiar and unfamiliar voices should be small, if evident at all.

To date, only two studies provide data on this point, yielding inconsistent findings (Cook & Wilding, 1997; van Lancker & Kreiman, 1987). Cook and Wilding (1997) examined the performance of 27 participants who completed a voice recognition task to both unfamiliar voices and to famous voices. The unfamiliar voice recognition task took the form of two lineup trials, in which participants made a selection from a six-alternative target-present lineup one week after initial exposure to the two target voices. The famous voice recognition task took the form of either a famous voice naming task to 6 celebrity voices (Experiment 1) or a famous/non-famous decision to 14 voices (7 celebrities) (Experiment 3). When correlated, the results suggested a significant correlation overall between unfamiliar voice recognition in the lineup task, and famous voice naming (Experiment 1: p < .05), which reached significance in only one of the three experimental conditions under scrutiny (short unvaried unfamiliar speech; *r* = .31, *p* < .05). In contrast, a non-significant correlation emerged when unfamiliar voice recognition was compared with the famous/non-famous decisions (Experiment 3: *r* = .29, *ns*) where arguably, the task demands for famous and for unfamiliar voice processing were better matched.

The results here are unclear for several reasons arising possibly from the relatively small number of participants, the use of just two lineup trials to determine unfamiliar voice performance, and the use of just 6 or 7 celebrity voices to determine famous voice recognition performance. In this regard, the results of van Lancker and Kreiman (1987) may be more robust. Whilst not perhaps the main focus of their paper, the authors tested 48 healthy participants who acted as controls alongside a cohort of 45 brain-damaged participants. The healthy controls completed a famous voice recognition test with 25 male celebrity voices, in which participants were asked to pair a 4 second clip of each voice with one of four face/name combinations. They also completed an unfamiliar voice discrimination test with 26 pairs of speech clips, uttered by 10 unfamiliar male speakers. The data suggested that both tasks could be completed at above-chance levels (familiar voice recognition = 82.1% accuracy; unfamiliar voice discrimination = 87.2% accuracy). However, somewhat surprisingly, performance across both tasks showed a moderate but significant correlation (*r* = .41, *p* < .01). Given the preceding discussion, a correlation in either of the studies, however moderate, was unanticipated.

The present study was designed to re-examine this issue. A familiar voice recognition task was used which took the form of a 2AFC (celebrity or not) decision to thirty 8-second speech clips. In addition, an unfamiliar voice discrimination task was used which took the form of a same/different matching task to 40 voices. The purpose of this study, therefore, lay in a re-exploration of the moderate correlations between familiar and unfamiliar voice processing revealed in Cook and Wilding’s (1997) Experiment 1, and in van Lancker and Kreiman’s (1987) control participants. On the basis of the review of evidence, it was anticipated that, whilst the two tasks would be possible at above-chance levels, performance across the tasks would not be correlated.

Method

*Design*

A repeated-measures design was used in which participants completed both a famous voice recognition task, and an unfamiliar voice discrimination task. The famous voice recognition task consisted of a famous/unfamiliar judgement to celebrity and non-celebrity voices. The unfamiliar voice discrimination task consisted of a same/different judgement to sequentially-presented pairs of unfamiliar voices. In both cases, accuracy of performance represented the dependent variable. In addition, in the famous voice recognition task, participants were asked to provide either a name or unique identifying information for any voices that they reported as familiar.

*Participants*

A total of 51 participants (36 females) took part on a volunteer basis or in return for course credit. Ages ranged from 18 to 40 years (*M* = 21.41, *SD* = 5.08) and all had normal, or corrected-to-normal hearing. The demographics of the participant group were constrained to include native English speakers, or those who had resided in the UK for a minimum of 7 years, ensuring that any task difficulties could not be attributable to a difficulty in speech comprehension. In addition, all participants were unfamiliar with all voices used in the unfamiliar voice matching task, as indicated by self-report at the end of that task.

*Materials*

*Familiar Speakers:* Stimuli were drawn from the internet and comprised a single voice clip of 15 adult celebrities (5 females, 10 males, actual ages at time of recording unavailable). The celebrities were all famous through stage or screen, and were selected on the basis of the familiarity of their voices according to a panel of eight judges drawn from the same population as the participants. Averaged ratings of celebrity familiarity ranged from 3 to 6.75 on a 7-point scale (*M =* 5.18, *SD* = 1.19), and all of the judges indicated familiarity with all of the voices and showed good inter-rater agreement (Cronbach’s Alpha = .84).

The celebrity speech clips consisted of 8 seconds of free speech within an interview or chat-show context in which the celebrity voice was the only voice heard. Care was taken to ensure that the speech clips were free from identifying information, as shown by the fact that a separate panel of eight judges was unable to name any of the celebrities from a transcript. As such, the clips conformed to the standards set by Schweinberger *et al.* (1997).

Accompanying the celebrity voices were the voices of 15 non-celebrity speakers. These were again drawn from the internet and comprised a single 8 second voice clip of a speaker within an interview context. The topics discussed in the interviews were such that the non-celebrity clips could not easily be distinguished from the celebrity clips. However, none of the speakers were recognised figures in the public eye. As with the celebrity speakers, all non-celebrities were Caucasian and of adult age, although actual age was not available.

All speakers spoke in English with either a British or an American accent, and the balance of accents was broadly matched across the celebrity and the non-celebrity speakers.

*Unfamiliar Speakers*: Unfamiliar voice stimuli were drawn from the SuperIdentity Stimulus Database ([www.soton.ac.uk/superidentity](http://www.soton.ac.uk/superidentity)) which provided a total of 121 speakers each providing multiple scripted voice clips. For the purposes of the unfamiliar voice matching task, the voices of 60 individuals were selected. Forty of these represented the target speakers and each provided two scripted speech clips. This enabled a different clip to be presented at study and at test in ‘same’ trials thus removing the potential for the matching task to be completed on the basis of clip-related characteristics alone. The remaining 20 speakers represented the foil speakers for use in ‘different’ trials, and these provided one scripted speech clip for use at the test stage. The clips themselves lasted 4-5 seconds in length, with a 500 msecs period of silence at the start of the clip to avoid an abrupt onset.

In terms of their characteristics, the targets were selected on the basis of their distinctiveness ratings (out of 7), as determined by a third panel of judges prior to the experiment. Agreement across the judges was excellent (Cronbach’s Alpha = .95). As a result, 20 voices (10 males, 10 females) were distinctive (*M =* 6.44, *SD* = .39) and 20 voices (10 males, 10 females) were typical (*M* = 2.27, *SD* = .55) and these groups differed significantly from one another in terms of distinctiveness (*t*_(34.22)_ = 27.52, *p* < .001). This ensured that the unfamiliar speakers spanned a range of vocal characteristics, providing generalisability to the task. The 20 foils (10 males, 10 females) were selected to be intermediate in terms of distinctiveness (*M* = 4.32, *SD* = .34), and they differed significantly in this regard from both the distinctive targets (*t*_(38)_ = 18.36, *p* < .001) and the typical targets (*t*_(31.33)_ = 14.18, *p* < .001). This ensured that the foil voices bore an equivalent similarity (or dissimilarity) to both the distinctive and typical targets, thus balancing the difficulty of the matching task across the two sets of stimuli.

Stimuli were combined to form 20 ‘same’ trials and 20 ‘different trials. The ‘same’ trials consisted of a target speaker uttering phrase 1 (‘The length of her skirt caused the passers-by to stare’) at study, and phrase 2 (‘They launched into battle with all the forces they could muster’) at test. The ‘different’ trials consisted of the target speaker uttering phrase 1 at study and a sex-matched foil uttering phrase 2 at test. Care was taken to counterbalance the items across the ‘same’ and ‘different’ trials across participants.

*Stimulus Presentation*: Stimuli were presented, and data were recorded via Superlab 4.5.4 running on a Hewlett Packard 350 G2 laptop running Windows 10. All instructions were presented via the 15.6” laptop screen, and sound clips were played via the computer speakers, with the volume pre-set to a comfortable yet adjustable level.

*Procedure*

Following the provision of informed consent, participants were tested individually within a quiet testing room. Participants were randomly assigned to one of two testing groups which differed only in the order of completion of the two tasks. This merely ensured that fatigue effects could not account for a difference in task performance. Regardless of task order, the format of the tasks was identical across the two groups as follows.

*Famous Voice Recognition Task:* The famous voice recognition task consisted of a practice phase, followed by a test phase. During the practice phase, participants were presented with 10 trials depicting the word ‘FAMOUS’ or ‘UNFAMILIAR’ in the centre of the screen. Their task was to press ‘F’ in response to the word ‘FAMOUS’ and to press ‘U’ in response to the word ‘UNFAMILIAR’. Feedback was provided for both correct and incorrect responses, encouraging the participants to map the correct response keys to each response.

Following this, the main task was administered and no further feedback was provided. The main task involved the presentation of 30 trials (15 celebrity, 15 non-celebrity) in a random order. Each trial followed an identical format consisting of a ‘ready…’ prompt for 500 msecs, and then a ‘famous or not?’ prompt question. This question remained on screen during the presentation of either a celebrity or a non-celebrity voice. Participants indicated whether the voice was famous (‘F’) or unfamiliar (‘U’) by pressing the appropriate key on the keyboard. If the voice was reported to be famous, participants were prompted for a name, or for other unique identifying information such as a character name, or the name of a spouse.

*Unfamiliar Voice Discrimination Task:* The unfamiliar voice discrimination task again consisted of a practice phase followed by a test phase. During the practice phase, participants were encouraged to press ‘S’ in response to the word ‘SAME’ and ‘D’ in response to the word ‘DIFFERENT’ as they appeared on screen. Following this, a second practice task introduced participants to the format of the experimental trials by asking them to respond ‘same’ (S) or ‘different’ (D) to two sequentially presented beeps. Feedback on both practice tasks was again provided to encourage the participants to map the correct response keys to each response.

Following these practice tasks, the main task was administered and no further feedback was provided. The main task took the form of a sequential same/different discrimination task in which a ‘ready…’ prompt was provided for 500 msecs, followed by a brief interval of 100 msecs. Then, the first voice was presented lasting 4-5 seconds. To make sure that the discrimination task was not too easy, a 5 second delay followed, before the presentation of a ‘same or different?’ prompt. This remained in view during the presentation of a second voice for 4-5 seconds. Following presentation, participants indicated whether the second voice was the ‘same’ (‘S’) speaker, or a ‘different’ (‘D’) speaker to the first. The two tasks together lasted approximately 20-25 minutes, after which participants were thanked and debriefed.

Results and Discussion

Given the use of a dichotomous response format for both the famous voice recognition task and the unfamiliar voice discrimination task, the accuracy scores for both tasks were converted using the signal detection framework (Green & Swets, 1966) to provide measures of sensitivity of discrimination (*d’*) and response bias (*C*). These two measures represented the main dependent variables.

Preliminary examination of *d’* revealed two participants who emerged as outliers in the unfamiliar voice discrimination task (according to a criterion of 1.5 x interquartile range above Q3 and below Q1). This suggested either a misunderstanding of the instructions, or an inability to complete the task. These two participants were dropped from all subsequent analyses.

*Sensitivity of Discrimination*

Sensitivity of discrimination is summarised in Table 1 for both the famous voice recognition task, and the unfamiliar voice discrimination task. One-sample *­t-*tests provided a comparison to the chance level of zero and revealed that performance in both tasks was significantly and substantially better than chance (famous: *M* = 1.62, *SD* = .98, *t*_(48)_ = 11.51, *p* < .001; unfamiliar: *M* = 2.67, *SD* = .79, *t*_(48)_ = 23.65, *p* < .001). In addition, performance in the famous voice recognition task suggested that 41% (*SD* = .26) of the voices correctly identified as famous could also be correctly named or identified by means of unique identifying information.

(Please insert Table 1 about here)

*Response Bias*

Response Bias (*C*) is summarised in Table 1, for both the famous voice recognition task and the unfamiliar voice discrimination task. Comparison to zero suggested a significant response bias in both tasks, with means indicating a conservative bias (to say ‘non-celebrity’) in the famous voice recognition task (*M =* .23, *SD* = .54; *t*_(48)_ = 2.96, *p* = .005) and a liberal bias (to say ‘same’) in the unfamiliar voice discrimination task (*M* = -.18 *SD* = .49, *t*_(48)_ = -2.56, *p* = .014).

*Correlation between Famous and Unfamiliar Vocal Identity Processing*

Of most importance was the exploration of any correlation between performance in the famous voice recognition task and the unfamiliar voice discrimination task. To this end, a Pearson’s bivariate correlation was conducted using *d’* in the two tasks as the measure of interest. This revealed no evidence of an association between the performance with familiar voices and that with unfamiliar voices (*r*_(49)_ = .12, *p* = .426). This offered tentative support for the prediction that familiar and unfamiliar voice processing relied on separate mechanisms.

The lack of a correlation here, whilst anticipated, did deviate from the moderate correlation reported by van Lancker and Kreiman (1987), and it is prudent to consider reasons for this difference. First, it is notable that the nature of the famous voice recognition task differed between van Lancker and Kreiman’s study and the present one in that van Lancker and Kreiman’s participants were required to match the familiar voice to one of four face/name combinations whereas the present participants made a famous/unfamiliar decision to celebrities and non-celebrities. This methodological difference involved a shift from a 4AFC response to a 2AFC response, but also involved a shift from a 1:4 task to a 1:N task which may be considered closer to the task of recognising a familiar voice in the real world (i.e., is that someone I recognise given all the voices I know?). Additionally, van Lancker and Kreiman’s participants only listened to celebrity voices, whereas the current participants were required to distinguish between celebrity and non-celebrity voices. Exploration of performance in the current task using famous voices only, again revealed a non-significant correlation with performance in the unfamiliar matching task (r_(49)_ = .07, *p* = .636) suggesting that the mere inclusion of responses to non-celebrity voices here did not account for the discrepancy in results. Nevertheless, it was possible that their inclusion in the task may have engendered a shift in task demands.

Perhaps more pertinent is a difference in the nature of the unfamiliar voice discrimination task across the two studies. Van Lancker and Kreiman’s task involved the discrimination of 26 pairs of voices uttered by 10 unfamiliar males with the result that some speakers must have been repeated across trials. The design thus offered the opportunity to develop some level of familiarity with some voices meaning that the unfamiliar voice discrimination task may not have reflected the processing of wholly unfamiliar voices. This was corrected here by using 60 unfamiliar voices, none of which were repeated across trials. Arguably, these two factors may have provided a purer test of familiar and unfamiliar voice processing as a basis for the present correlational analysis.

*A Note of Caution:*

Whilst the lack of a significant correlation between the processing of familiar and unfamiliar stimuli was to be expected, some caution must be exercised in the interpretation of the current data. Correlations of performance across tasks have been conducted in the published literature (i.e., Cook & Wilding, 1997; van Lancker & Kreiman, 1987), and the present study was undertaken with a replication of that approach in mind. Nevertheless, many things varied between the two tasks other than stimulus familiarity (including possibly the distinctiveness or variability of the stimuli themselves, and the task demands associated with the two quite different tasks). This makes it difficult to conclude that the lack of any correlation here was due to stimulus familiarity alone.

A better approach may be to adopt a battery of tests examining familiar voice processing and unfamiliar voice processing with the prediction of a stronger correlation between the tests measuring performance within familiarity, than between those measuring performance across familiarity. Work along these lines is ongoing.

Setting aside a correlational approach, a far stronger empirical approach would involve a task which can be conducted with familiar and unfamiliar voices alike so that task demands can be aligned across the familiar and unfamiliar tests. The most elegant way to do this would be to use a single set of stimuli with which one group of listeners is familiar, and another group of listeners is not. In this way, stimulus characteristics can be held constant whilst familiarity is varied. The study reported in the main paper provides exactly this approach, and uses a voice sorting task with voices that are either personally familiarity or unfamiliar to the listeners.

Table 1:

Mean sensitivity of discrimination (d’) together with response bias (C) for the famous voice recognition task and the unfamiliar voice discrimination task. (Values in parentheses indicated standard deviation.)

|  | Famous Voice Recognition | Unfamiliar Voice Discrimination |
| --- | --- | --- |
| Sensitivity of Discrimination (d’) | 1.62 (.98) | 2.67 (.79) |
| Response Bias (C) | .23 (.54) | -.18 (.49) |
